# Assessment of the Sterility of New Endodontic Files Received From the Manufacturer Using Microbial Culture and Scanning Electron Microscopic Analysis: An In Vitro Study

**DOI:** 10.7759/cureus.28092

**Published:** 2022-08-17

**Authors:** Khalid A Merdad, Faisal T Alghamdi

**Affiliations:** 1 Endodontics, Faculty of Dentistry, King Abdulaziz University, Jeddah, SAU; 2 Oral Biology, Faculty of Dentistry, King Abdulaziz University, Jeddah, SAU

**Keywords:** sterilization, cleaning, infection control, new files, endodontic files

## Abstract

Background

As endodontic hand files are essential tools for root canal instrumentation and treatment, these instruments have been a constant source of debate regarding whether to reuse them or use them just once. The aim of this in vitro study is to evaluate the sterility of five brands’ new endodontic files received from manufacturers without sterilization before clinical use via microbial culture (bacteria and fungi) and bioburden using scanning electron microscopic (SEM) analysis.

Methodology

This study included 25 K-type hand files with equal numbers from five brand groups (n=5; groups 1 to 5), named Tia Dent, Prevalent, Mani, Thomas, and Dentsply, respectively. The new files were exposed to microbial culture prior to clinical use and SEM analyses. A Fisher’s exact test was performed to compare the differences in contamination among the five tested groups.

Results

In the SEM, all the five brand groups showed one contaminated file out of five files (20%). Fisher's exact test findings revealed statistically significant variations between the five brand groups, as nine out of 25 (36%) files of the tested groups had positive contamination in the microbial culture experiment. Bacterial contamination was present in three (60%) of the five Tia Dent group files, four (80%) in the Prevalent group files, and two (40%) in the Mani group files. Those with fungal contamination were one (33%) in the Tia Dent group and one (25%) in the Prevalent group (P=0.027). The bacterial culture showed that 100% of the new K-files tested negative in both the Thomas and Dentsply groups.

Conclusions

This study concluded that there was a bioburden on new endodontic K-type hand files of different brand groups before sterilization or when immediately used.

## Introduction

Successful endodontic therapy is dependent on the eradication of microorganisms from the root canal as well as the prevention of re-infection [1]. Therefore, it is crucial to establish and maintain an aseptic chain during endodontic therapy [2]. Endodontic files are important tools for instrumenting the root canal system. To avoid a potential break in the aseptic chain and eventual treatment failure, these tools must be free from microbes before being inserted into the root canal [1]. Additionally, they are classified as “critical” when they contact sterile body regions, penetrate oral mucosa, or enter the blood circulation [3].

Several studies were performed to test the sterility and the presence of debris in new, unused endodontic files received from manufacturers. They indicated that none of the instruments were considered clean or sterile [4-8]. Roth et al. found biological contamination in one out of six files received from the manufacturers [9]. Moreover, Zmener and Speilberg devised a process for cleaning pre-utilization for patients because all the unused and new files were contaminated when examined [5].

According to the guideline for disinfection and sterilization in healthcare facilities, the instruments are categorized as critical, semi-critical, and non-critical according to the level of risk for infection involved in the use of these instruments [10]. Items that come in contact with the body, penetrate the oral mucosa, or enter the bloodstream are categorized as “Critical Items” and must be sterilized before use [3]; these instruments include hand and rotary files, endodontic surgical instruments, burs and periodontal probe. Semi-critical items are defined as instruments that do not penetrate the vascular system but are used inside the mouth and contact with the mucosa, such as impression tray. The non-critical items include items that do not contact body fluids or intact skin, such as radiographic equipment, facebows, pulse oximeters, examination and curing lights, and plastic filling instruments that are recommended to be disinfected. In endodontics, the root canal space is considered as vascular system and all the instruments used are considered as critical items [10].

Some files come in sterilized packages with clear signs and expiry dates for sterilization, and many file packages don’t reveal any claims or complaints of product sterility. Several clinicians might think a new file is sterile and may use it directly on a patient prior to sterilization.

A review of the literature revealed that no studies had been done to assess whether or not microbial contamination and debris were present in the wide range of new, unused files that had been received from the manufacturer in Saudi Arabia. The purpose of this in vitro study is to evaluate the sterility of five brands’ new endodontic files received from manufacturers without sterilization before clinical use via microbial culture (bacteria and fungi) and biological debris contamination using scanning electron microscopic (SEM) analysis.

## Materials and methods

This in vitro investigation was conducted at the Faculty of Dentistry at King Abdulaziz University. The Faculty of Dentistry at King Abdulaziz University's Research Ethics Committee gave its approval to the study protocol (Approval No. 314-11-21). This type of investigation does not need informed consent. A total of 25 endodontic hand files of stainless-steel K-type were included in this in vitro study, and they were categorized with equal numbers in five groups of K-file brands. The K-file size was 40 with a length of 25mm. The files were collected from different brands that are commonly used in the Saudi market in Jeddah, Saudi Arabia, and a simple random sampling technique was used in this study. This study is in compliance with the World Medical Association Declaration of Helsinki providing ethical practices in experiments. For this experiment, we collected and randomly allocated five brands of K-type hand files into groups which are as follows: Group 1: Tia Dent (Tia Dent Corp., Huston, TX, USA) (n=5); Group 2: Prevalent (Prevalent, Guangming, Shenzhen, China) (n=5); Group 3: Mani (Mani, Tochigi, Japan) (n=5); Group 4: Thomas (French Dental Products, Société-FFDM Pneumat, Département Dentaire Thomas, Bourges Cedex, France) (n=5); and Group 5: Dentsply (Dentsply Maillefer, Ballaigues, Switzerland) (n=5). The authors achieved a power of 0.80 with an alpha (α) level of 0.05 (Confidence level: 95%), and a sample size of 25 was considered for the total sample [11].

Scanning electron microscopic (SEM) analysis

**Figure 1:**
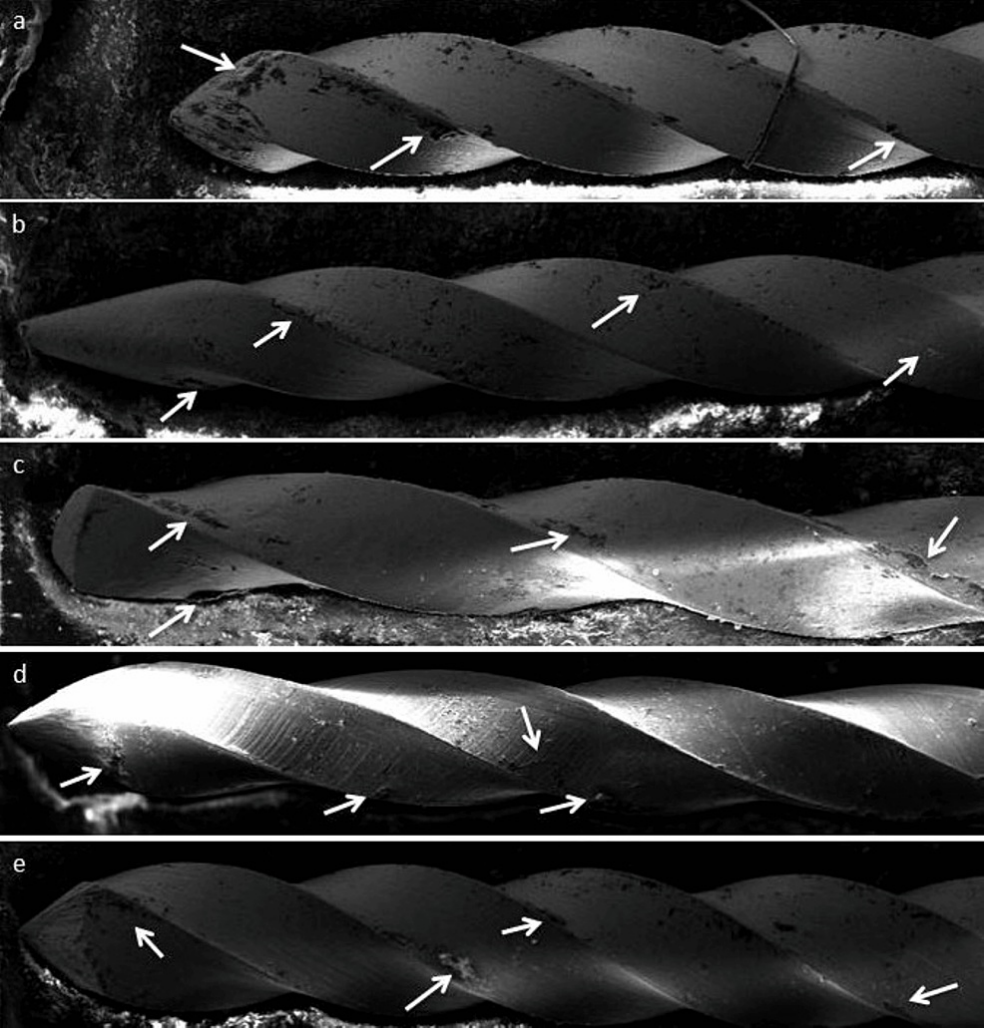
SEM of new endodontic K-files. Observe clear surfaces on files that have small metallic fragments, surface defects, and debris (pointed by arrows) in (a) TiaDent file, (b) Prevalent file, (c) Mani file, (d) Thomas file, and (e) Dentsply file. SEM: Scanning Electron Microscopy

All the files in each of the five brand groups were photographed by the SEM. The photographs were captured and a 40x magnification and a 15 kV accelerating voltage were used to project them onto a computer. The new files also served as controls to denote the normal appearance. SEM (Germany's Zeiss Q150R) was used to observe the small metallic fragment, surface defects, and bioburden on the files at the flutes and cutting surfaces (Figure 1). SEM photographs were examined by two examiners (authors of the article).

Microbial analysis

**Figure 2:**
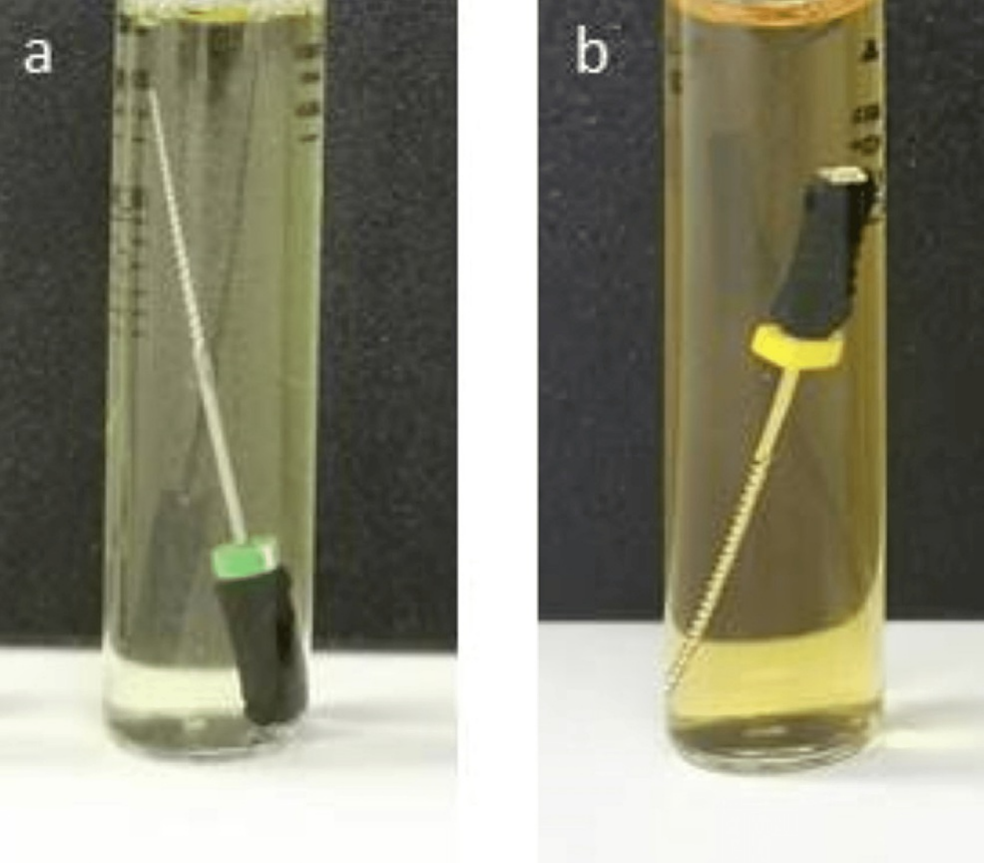
New endodontic files are cultured in tubes (10 mL) of TSB broth. One of the control endodontic K file samples was free of turbidity (negative control sample). (a) A positive control sample represents a contaminated used file without sterilization; (b) A negative control sample represents a new file after the sterilization. TSB: trypticase soya broth

**Figure 3:**
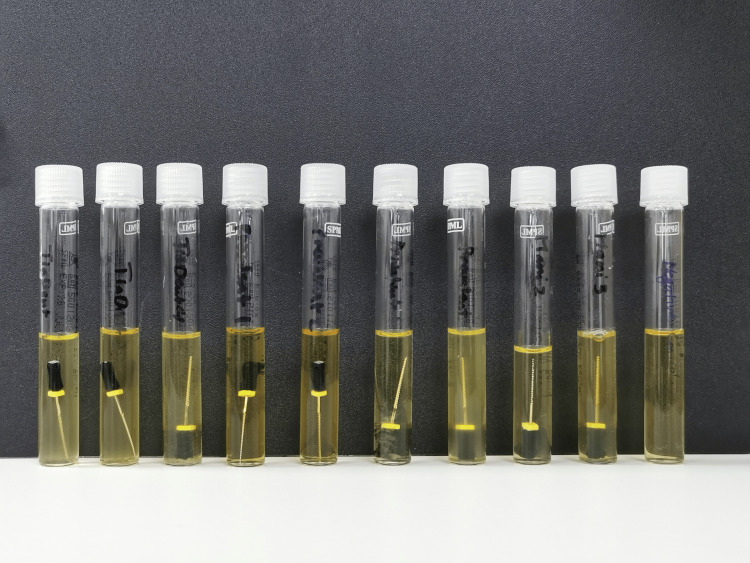
New endodontic files are cultured in tubes (10 mL) of TSB. In comparison to a clear (negative) control tube with just broth, all of the endodontic K files exhibited turbidity. TSB: trypticase soya broth

Bacterial culture was performed on all 25 K-type hand files, which were aseptically inoculated in sterile test tubes with 10 ml trypticase soya broth (TSB). The TSB is a fastidious organisms-specific growth medium. For this culture, negative control with an extra test tube with just 10ml TSB was set up. To avoid any contamination during the experiment, sample collection was done in a strictly sterilized approach. In addition, to ensure the validity of the test tube by TSB, we selected two files randomly from the available endodontic hand K-files in the Saudi dental market as control groups (one positive control sample represents contaminated used files without sterilization and one negative control sample represents new files after sterilization) before starting the experiment (Figure 2). For 10 days, all of the tubes were cultured at 37°C in a 5% CO2 setting. The presence of microbial colonies was determined by measuring the turbidity in the test tubes (Figure 3).

**Figure 4:**
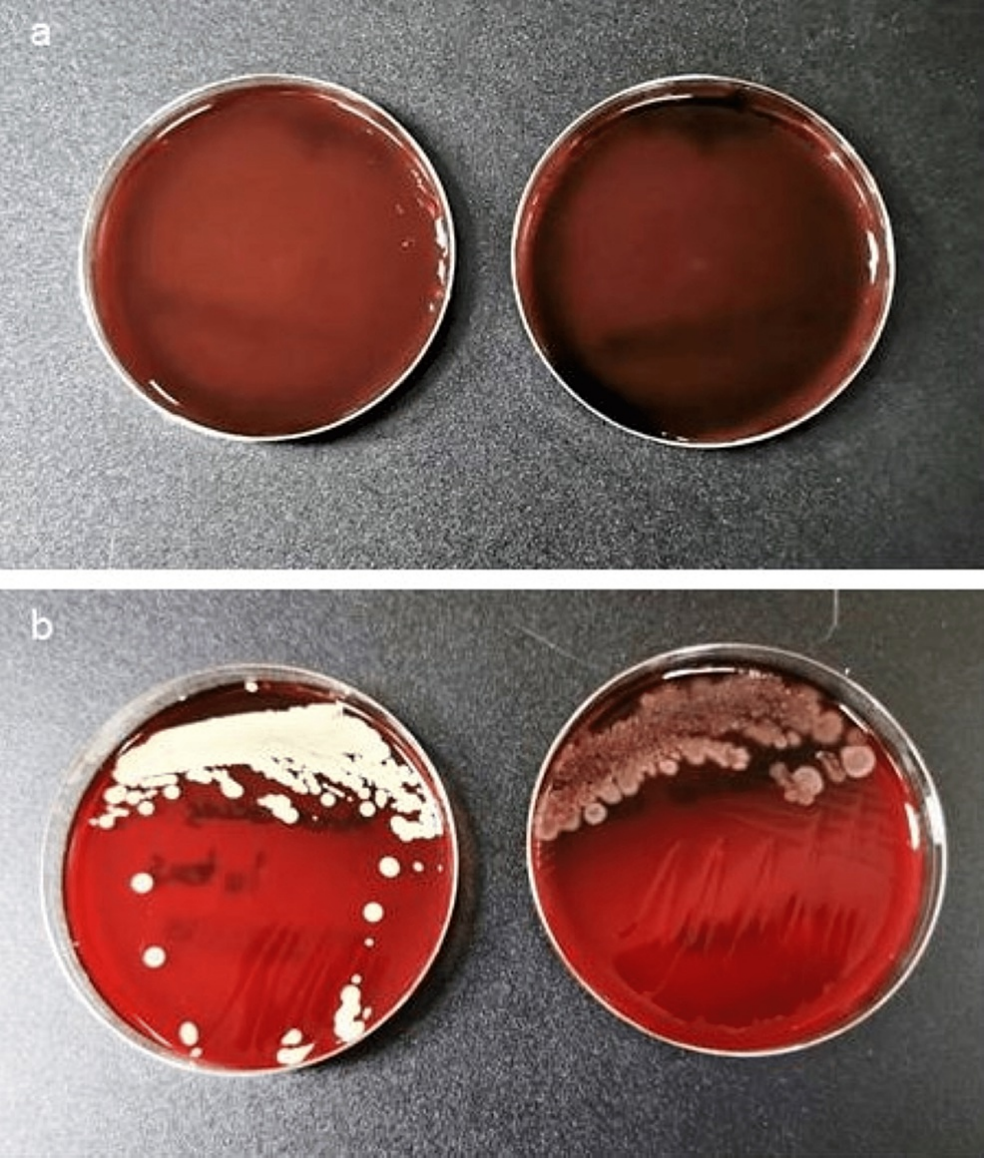
Culture plates of the new K files. (a) A representative plate with no growth in new K files. (b) Bacterial growth in new K files.

The turbidity-tested test tubes were used to obtain an inoculum, which was then introduced to culture plates (blood agar plate; BAP) containing TSB and sheep blood in trypticase soy agar (TSA) (Remel Product, Thermo Fisher Scientific, Lenexa, KS, USA). A general-purpose medium that is used for yeasts and molds is potato dextrose agar (PDA). The blood agar plates were cultured in 5% carbon (IV) oxide at 37°C for more than 48 hours. For five days, the BAP with the inoculum was cultured at 300°C, and the procedure was repeated to produce a pure culture of colonies. The BAP was then kept in 20% glycerol at -80°C (Figure 4). Gram staining and other biochemical tests, such as growth in alkaline media and the catalase test, were used to determine the identification and characteristics of the bacterial colonies (pH of 9.2). The Microbiology Powered by Mass Spectrometry (VITEK® MS) (bioMérieux, Inc., Durham, NC, USA), an automated identification system, was employed that allows for rapid identification of the bacteria. Three different media (Capek's solution, malt extract agar, and PDA) were used to cultivate the fungal growth that was seen in the Petri plates over seven days at 300°C unless pure cultures were collected and preserved. The morphology of the fungus including its surface or front appearance and color was used to identify it.

Statistical analysis

The collected data were analyzed using SPSS Version 20.0 for Windows (IBM Corp., Armonk, NY, USA). The data were presented as frequencies and percentages. A Fisher’s exact test was performed to compare the differences in contamination among the new K-hand files samples, and differences between the five brand groups of K-type hand files were also evaluated. For a test, a P-value of ≤0.05 was considered to be significant statistically.

## Results

The present study was performed on new K-type endodontic hand files (n=25), and equal number of files were divided among the five groups. The frequency distribution in this study showed that there were 20% K-type hand files belonging to five brand groups (Table 1). The SEM images for all the brand groups of files showed that only one file (20%) was contaminated with biological debris in each brand group (Table 2 and Figure 1).

**Table 1:** Distribution of new K-type of hand files among the five brand groups.

Study Samples	Tia Dent, N (%)	Prevalent, N (%)	Mani, N (%)	Thomas, N (%)	Dentsply, N (%)	Total, N (%)
	5 (20)	5 (20)	5 (20)	5 (20)	5 (20)	25 (100)

**Table 2:** Contamination with biological debris among the new K-type hand files in the five brands under SEM. SEM: Scanning Electron Microscopic

Brand	Contaminated with biological debris, n (%)	
	NEGATIVE	POSITIVE
Tia Dent (n=5)	4 (80)	1 (20)
Prevalent (n=5)	4 (80)	1 (20)
Mani (n=5)	4 (80)	1 (20)
Thomas (n=5)	4 (80)	1 (20)
Dentsply (n=5)	4 (80)	1 (20)
Total (n=25) n (%)	20 (80)	5 (20)

The research was conducted in multiple parts to evaluate the bacterial and fungal contaminations on new K-hand files using a microbial culture. The bacterial culture results showed that nine out of 25 samples (36%) tested positive for contamination in the following groups: three (60%) in the Tia Dent group, four (80%) in the Prevalent group, and two (40%) in the Mani group with P=0.027 (Table 3), while two groups tested negative (no contamination) in their samples for bacterial and fungal contaminations. Regarding these bacterial culture results, there were statistical significances among the five tested groups (P=0.027) (Table 3).

**Table 3:** Presence of bacteria or fungi among the new K-type hand files in the five brands. *Using Fisher’s Exact Test.

Brand	Bacterial / Fungal Contamination n (%)	
	NEGATIVE	POSITIVE
Tia Dent (n=5)	2 (40)	3 (60)
Prevalent (n=5)	1 (20)	4 (80)
Mani (n=5)	3 (60)	2 (40)
Thomas (n=5)	5 (100)	0 (0)
Dentsply (n=5)	5 (100)	0 (0)
Total (n=25) n (%)	16 (64)	9 (36)
P-value*	0.027	

The comparisons in positive bacterial contamination among the tested groups showed contamination in seven (78%) out of nine samples in the following groups: two (67%) in the Tia Dent group, three (75%) in the Prevalent group, and two (100%) in the Mani group (Table 4). On the other hand, the comparisons in positive fungal contamination among the tested groups showed fungal contamination in two (22%) out of nine samples in the following groups: one (33%) in the Tia Dent group and one (25%) in the Prevalent group (Table 4).

**Table 4:** Frequencies and percentages of the bacterial and fungal positive samples among the new K-type hand files in the five brands.

Brand	Bacterial	Fungal
	POSITIVE n (%)	POSITIVE n (%)
Tia Dent (n=3)	2 (67)	1 (33)
Prevalent (n=4)	3 (75)	1 (25)
Mani (n=2)	2 (100)	0 (0)
Total (n=9) n (%)	7 (78)	2 (22)

**Figure 5:**
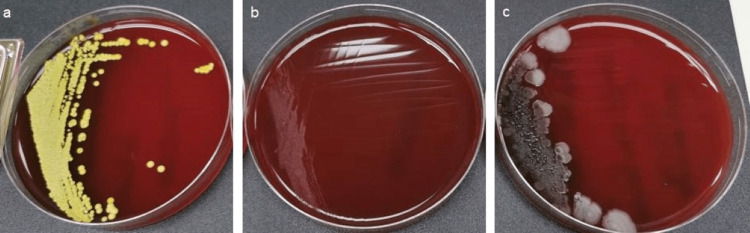
Culture plates of the new K files in the Tia Dent group. Representative culture dish showing (a) Micrococcus luteus, (b) fungal growth (yeast), and (c) Bacillus subtilis.

**Figure 6:**
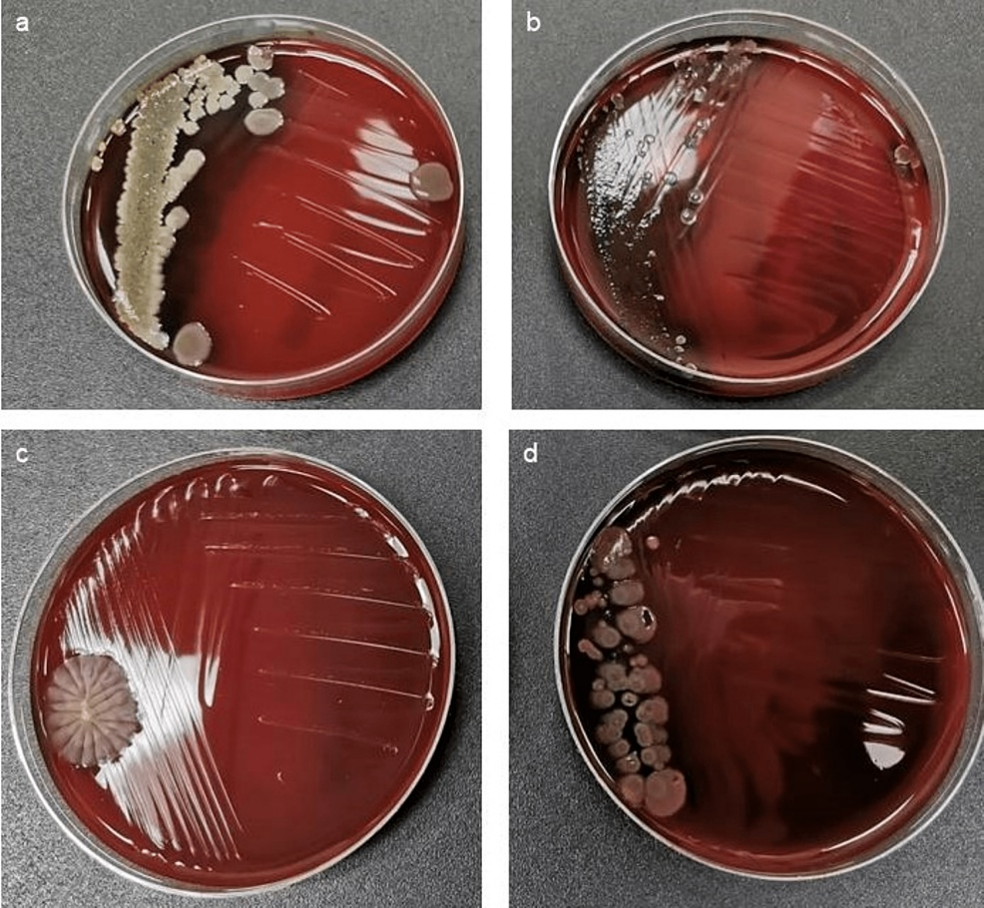
Culture plates of the new K files in the Prevalent group. Representative culture dish showing (a) Bacillus firmus, (b) Bacillus subtilis, (c) fungal growth (mold), and (d) Bacillus unknown species.

**Figure 7:**
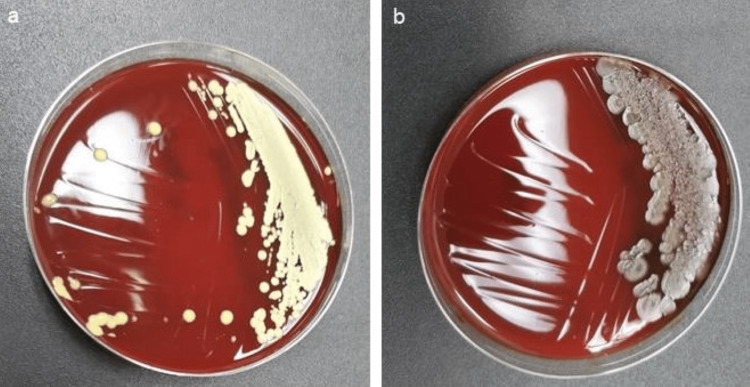
Culture plates of the new K files in the Mani group. Representative culture dish showing (a) Micrococcus luteus and (b) Bacillus firmus.

The bacterial species identified from three contamination groups are *Micrococcus luteus *(*M. luteus*), *Bacillus subtilis* (*B. subtilis*), *Bacillus firmus* (*B. firmus*), and *Bacillus *unknown species (Figures 5-7), whereas yeast and mold as fungal contamination samples were isolated from two contamination groups (Figures 5, 6). The bacterial species identified were distributed as follows: two samples in each group from the following species: *M. luteus* (Tia Dent and Mani groups), *B. subtilis* (Tia Dent and Prevalent groups), and *B. firmus* (Prevalent and Mani groups), while only one sample came from *Bacillus* unknown species (Prevalent group). The fungal species identified were distributed as follows: one sample from yeast unknown species (Tia Dent group) and one sample from mold unknown species (Prevalent group). Table 5 provides the frequencies and percentages of bacteria and fungi types among the contaminated new K-type hand files.

**Table 5:** The frequencies and percentages of bacteria and fungi types among the contaminated new K-type hand files.

Brand	Bacteria				Fungi	
	Micrococcus luteus	Bacillus subtilis	Bacillus firmus	*Bacillus *unknown species	Yeast	Mold
Tia Dent (n=3) n (%)	1 (33.3)	1 (33.3)	0 (0)	0 (0)	1 (33.3)	0 (0)
Prevalent (n=4) n (%)	0 (0)	1 (25)	1 (25)	1 (25)	0 (0)	1 (25)
Mani (n=2) n (%)	1 (50)	0 (0)	1 (50)	0 (0)	0 (0)	0 (0)
Total (n=9) n (%)	2 (22.2)	2 (22.2)	2 (22.2)	1 (11.1)	1 (11.1)	1 (11.1)

**Figure 8:**
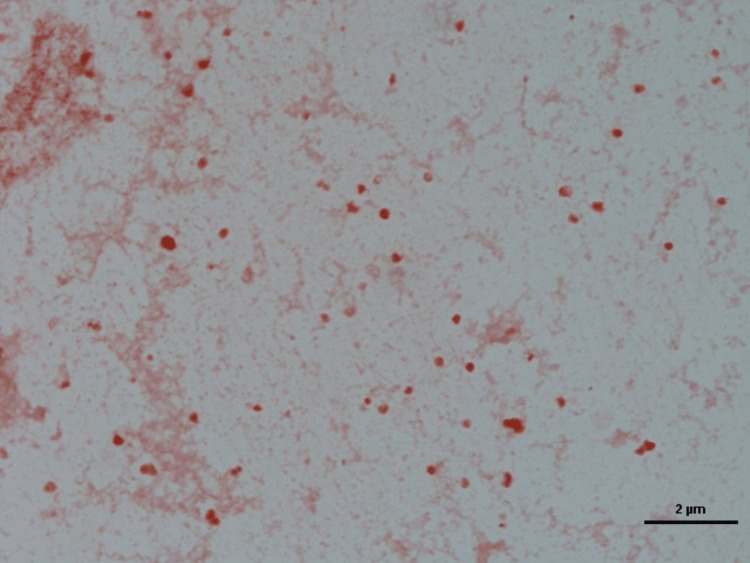
One colony-forming unit (CFU) of yeast isolated under light microscopy. Representative culture dish of Tia Dent group showing unknown species of fungi.

**Figure 9:**
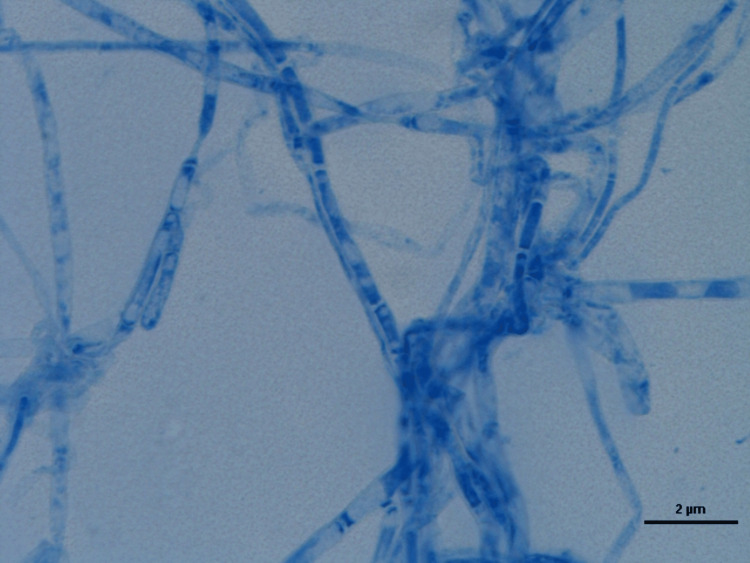
One colony-forming unit (CFU) of mold isolated under light microscopy. Representative culture dish of Prevalent group showing unknown species of fungi.

**Figure 10:**
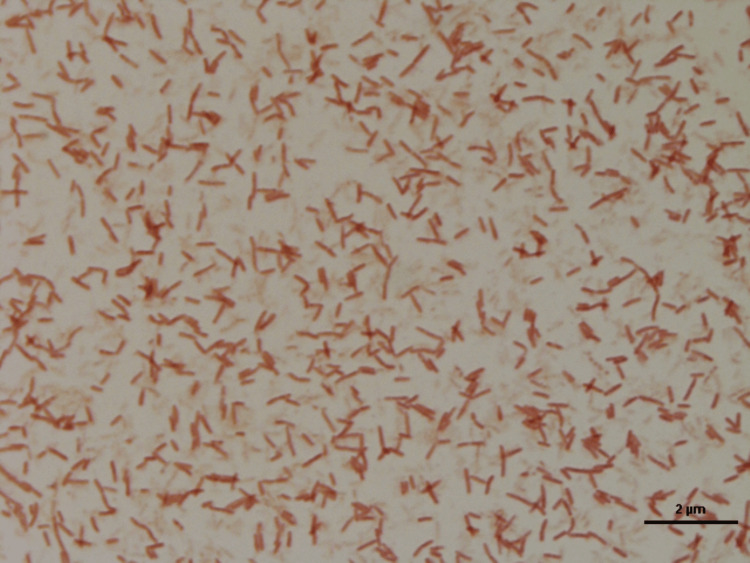
One colony-forming unit (CFU) of bacteria isolated under light microscopy. Representative culture dish of Prevalent group showing unknown species of bacillus.

Regarding the three unknown species samples of fungi and bacteria, one colony-forming unit (CFU) of yeast was isolated. The conventional method, macroscopic and microscopic findings, showed it was not identified as clinically significant fungi. So, it could be one CFU of environmental fungi (yeast) isolated (Figure 8). Also, one CFU of mold was isolated. The conventional method, macroscopic and microscopic findings, showed it was not identified as clinically significant fungi. So, it could be one CFU of environmental fungi (mold) isolated (Figure 9). On the other hand, the conventional method, analytical profile index (API), vitkMS, and Vitk 2 showed it was not identified as clinically significant bacteria. So, it could be environmental bacteria (*bacillus*) isolated (Figure 10).

## Discussion

Instruments that come in contact with the body, penetrate the oral mucosa, or enter the bloodstream are categorized as ‘Critical Items’ [10], therefore, following an aseptic technique in preparing a root canal system is crucial to the outcome of endodontic treatment and most importantly to the overall health of the patient [3]. In our study, the investigated endodontic instrument samples included hand K-files from different brand groups which were considered critical items only according to the guideline for disinfection and sterilization in healthcare facilities [10]. Thus, endodontic files should be sterilized before clinical use to prevent the transmission of infectious diseases in clinical practice [3].

Several investigations were targeted to investigate the sterility of packaged new endodontic files received from different manufacturers [9, 12-16]. A recently published investigation done by Abumelha et al. on the effectiveness and duration of endodontic files sterilization revealed that 14 (9%) (new unused endodontic files received from the manufacturer) of 158 files had tested positive for microbial culture before the sterilization [14]. Furthermore, Rajalakshmi and Jayalakshmi discovered that 12 out of 15 files received from the manufacturers were contaminated [16]. A previous investigation done by Roth et al. on the sterility of new unused endodontic files received from six manufacturers revealed that 13% of the 150 files had tested positive for microbial cultures [9]. Gnau et al. [12] reported a 6% rate of contamination while Morrison and Conrod found 45% contamination in endodontic files [13]. Murgel et al. [15] reported 10 unused, non-cleaned files as a negative control group, they were not completely free of debris. These results indicate that endodontic files are not sterile at the time of purchase and sterilization should be performed prior to first use [9, 12-15]. Failing to perform proper disinfection can lead to serious consequences. These results in agreement with our study results were conducted on 25 endodontic hand files that belonged to the K-type with a size of 40 and length of 25mm and equal numbers were present in the five brand groups for assessment of sterility of packaged new endodontic files. The results have proven the presence of biological debris and viable microorganisms on new endodontic files. Furthermore, the microbial analysis revealed that none of the new hand files of all the five brand K-file groups showed any evidence of bacterial or fungal contamination: two (40%) in the Tia Dent group, one (20%) in the Prevalent group, three (60%) in the Mani group, and five (100%) in both Thomas and Dentsply groups. On the other hand, three files (60%) in the Tia Dent group, four (80%) in the Prevalent group, and two (40%) in the Mani group tested positive for bacterial and fungal contamination, and it was statistically significant (P=0.027) (Table 3). Only one set of cultivation conditions was utilized to extract potential biological contaminants, and it is well known that the preponderance of bacterial species is presently not cultivable in the laboratory, therefore this estimate is most likely underestimated [17]. For the two brands (Thomas and Dentsply groups) that didn’t show bacterial contamination, it could be explained that certain file types were packed in sealed boxes, but most of the files weren't protected from the external environment by their packaging. As a result, contamination might have happened both throughout the manufacturing process and throughout the transfer from the manufacturer to the clinicians.

In the present investigation, the most prevalent microbial species isolated were *M. luteus*, *B. subtilis*, and *B. firmus* (Gram-positive) which have been linked with health issues through their colonization and infection in the oral cavity. From this study, it can be observed that pathogenic microorganisms were isolated from the three brands of K-file groups before sterilization or when immediately used for clinical practice. Therefore, it demands further analysis, including a microbial load evaluation and a larger sample size, to determine its clinical relevance.

This current study reported both bacterial and fungal contaminations as atmospheric microorganisms frequently present on environmental endodontic instruments. These are one of the most prevalent contaminants among factory cultures. These contaminations are frequently seen on agar plates during bioburden testing of dental instruments prior to sterilization.

Several studies [9, 12-15] showed different bacterial species among their tested packed new endodontic files including Gram-positive spore-forming rods and Gram-positive cocci, Gram-variable spore-forming rods, and Gram-negative bacteria. In contrast, our current study showed *M. luteus*, yeast, molds, and different species of the *Bacillaceae* family are the most prevalent microbes found among the tested endodontic hand K-file samples. Therefore, further investigation on many microbial types that may be found on various endodontic tools will be required in the future.

Most endodontic instruments supplied by manufacturers are not sterile and have been found to have metallic spurs, grease, epithelial cells, plastic, and debris on their surfaces [18, 19]. In these studies, all brands of files show evidence of biological debris. Debris may include dead cells and foreign particles which can trigger inflammation and foreign body reaction, even if they are sterile [20]. Biological waste or debris on equipment might prevent efficient sterilization and it is a widely accepted fact [21]. It may be caused due to the ineffectiveness of steam penetration or due to biological waste with minimal moisture content, which might make vegetative bacteria and spores more heat-resistant [22]. In a study done by Van Eldik et al. [23] in 2004, they found that no bacteria were detected in stainless steel and nickel-titanium (NiTi). No matter what kind of cleaning method was used before, NiTi files were treated with steam sterilization. In addition to this, the results of a recently published study showed negative tests for bacterial culture (100%) among new H&K-types hand endodontic files after sterilization [24]. Although bacterial growth on files taken from the manufacturer's packing is considered to be limited, Standards Australia [25] recommends that files be sterilized in suitable packaging before use to assure instrument's sterility. Thus, the ADA (American Dental Association) continues to recommend that dental equipment, particularly endodontic files, be removed of bioburden before sterilization [3].

Some files come in sterilized packages with clear signs and expiry dates for sterilization, and many file packages don’t reveal any claims or complaints of product sterility. Several clinicians might think a new file is sterile and may use it directly on a patient prior to sterilization. Other investigations have illustrated that after sterilization and packing, instruments may stay sterile for up to 12 months [26, 27]. Our study confirms the presence of microorganisms and biological debris in the new files received from manufacturers. This could be due to the manufacturing process that deals with raw materials, oil, milling machine, and handling process. Furthermore, the package which is commonly delivered in an unsealed box can create a favorable environment for bacteria to grow due to exposure to the external environment during transportation and storage [23, 28].

The effects of various cleaning techniques in NiTi rotary files were compared by Linsuwanont et al. [29]. They removed debris using different methods from 180 endodontic files (brushing, immersion in sodium hypochlorite (NaOCl) 1% for 10 minutes after brushing, immersion in NaOCl 10% for 10 minutes, ultrasonic for 5 minutes, the combination of all previous methods). Their findings demonstrated that it was more effective to combine all strategies [29].

As per our review of literatures, this in vitro study is the first research of its kind to investigate the presence or absence of both bacteria and fungi contamination in addition to the presence of biological debris under SEM among a wide variety of new unused files received from manufacturers in Saudi Arabia. This issue is vital and essential since some companies don’t declare sterility of new files and clinicians may assume product sterility and use it directly on patients.

This study has several limitations. Firstly, it is known that not all viable microbes are cultivatable and they may not grow under laboratory conditions [17, 30-33]. In the applicable scenario to this study, the new files received from the manufacturers may entertain many microorganisms (bacteria, viruses, and fungi) which can’t grow in a laboratory while they exist and can cause a potential risk of infection in humans. An example of the dormancy phase of bacteria has been explained for Gram-negative bacteria and termed as the viable but nonculturable (VBNC) [31]. Focusing on uncultivable bacteria, they are divided into two groups: bacterial groups with no cultivated representatives (yet-to-be-cultivated cells), and others belonging to groups that have been previously cultivated in the laboratory but whose cells are in a state in which they are alive but no longer replicating (non-dividing cells) [33]. Secondly, only aerobic bacteria were detected in this study due to the environment of these bacteria [32]. No anaerobic bacteria were observed in this experiment due to all the investigated samples were new packed endodontic files without sterilization before clinical use compared to sterilized/non-sterilized after clinical use which illustrated high evidence of anaerobic bacteria among the investigated endodontic files [23, 24]. Thirdly, only one size of K-file was investigated in this experiment; however, the size and design of the cross-section could contribute to the amount of debris retained during manufacturing between the flutes. Van Eldik et al. demonstrated that rotary files retained more bacteria when compared to the Hedström hand files [23]. Therefore, studying the sterility of different file designs including rotary instruments is essential.

The purpose of this investigation was not to identify the presence of living microorganisms and debris on new endodontic files, but instead to identify the type of bacteria present. This study can be taken as a preceding step for other studies centered around the bacterial evaluation of other armamentariums used in dentistry, that are directly used by the doctors on the patient (e.g., burs), and as a way to raise awareness for the sterilization of new unused endodontics instruments in the dental community.

## Conclusions

The present research concluded that the new endodontic files received from the manufacturer were contaminated and should be sterilized very well before clinical use. The results showed viable microbes (bacteria and fungi) contaminations in Tia Dent, Prevalent, and Mani groups only, while samples of Thomas and Dentsply groups were not detected with any bacterial or fungal contaminations. On the other hand, all the five brand groups showed evidence of biological debris under the SEM with one file (20%) from each of the five brand groups. The highest standards of infection control have to be maintained in routine dental practice and clinicians must clean and sterilize files before clinical use. Further research is warranted to evaluate the viral and other types of bacteria species present in packaged endodontic files and other endodontic instruments used during the endodontic treatment, that are directly used by the doctors on the patients, and to raise awareness for sterilization of packaged new unused endodontic instruments among the dental practitioners.
